# FGF8 and BMP2 mediated dynamic regulation of dental mesenchyme proliferation and differentiation via Lhx8/Suv39h1 complex

**DOI:** 10.1111/jcmm.16351

**Published:** 2021-02-13

**Authors:** Chen Zhou, Danying Chen, Jianhan Ren, Delan Huang, Runze Li, Haotian Luo, Chenyu Guan, Yang Cao, Weicai Wang

**Affiliations:** ^1^ Hospital of Stomatology Sun Yat‐sen University Guangzhou China; ^2^ Guangdong Provincial Key Laboratory of Stomatology Sun Yat‐sen University Guangzhou China; ^3^ Guanghua School of Stomatology Sun Yat‐sen University Guangzhou China

**Keywords:** BMP2, Differentiation, FGF8, Lhx8, Proliferation, Suv39h1

## Abstract

The homeobox gene, LIM‐homeobox 8 (Lhx8), has previously been identified as an essential transcription factor for dental mesenchymal development. However, how Lhx8 itself is regulated and regulates odontogenesis remains poorly understood. In this study, we employed an RNAscope assay to detect the co‐expression pattern of Lhx8 and Suv39h1 in the dental mesenchyme, which coincided with the dynamic expression profiles of the early epithelium signal of Fibroblast Growth Factor 8 (FGF8) and the later mesenchymal signal Bone Morphogenetic Protein 2 (BMP2). Moreover, FGF8 activated Lhx8, whereas BMP2 repressed Lhx8 expression at the transcriptional level. The high expression of Lhx8 in the early dental mesenchyme maintained the cell fate in an undifferentiated status by interacting with Suv39h1, a histone‐lysine N‐methyltransferase constitutively expressed in the dental mesenchyme. Further in the ex vivo organ culture model, the knockdown of Suv39h1 significantly blocked the function of Lhx8 and FGF8. Mechanistically, Lhx8/Suv39h1 recognized the odontoblast differentiation‐related genes and repressed gene expression via methylating H3K9 on their promoters. Taken together, our data here suggest that Lhx8/Suv39h1 complex is inversely regulated by epithelium‐mesenchymal signals, balancing the differentiation and proliferation of dental mesenchyme via H3K9 methylation.

## 1 INTRODUCTION

Tooth development begins from sequential and reciprocal signalling interactions from the stomadial epithelium and cranial neural crest‐derived mesenchymal cells.^1^ During the murine molar development, the dental epithelium thickens at embryonic day 11.5 (E11.5) and then invaginates into the underlying condensed mesenchyme, forming the tooth bud at E12.5‐E13.5. By E14.5, the tooth bud continues to the cap stage, and the tooth morphology is established.^2^ Recently, the molecular mechanisms at the transcriptional level that control tooth development are intensively studied, shedding light on the rule of development in both the tooth and the other organs.^3^

The interaction and subsequent morphological changes of dental epithelium and mesenchyme are accompanied and executed by activity changes in numerous genes, such as genes that encode growth factors, transcription factors and the extracellular matrix.^4^ The LIM‐homeobox 8 (Lhx8), which is also known as L3 and Lhx7, is a remarkably conserved transcriptional factor of the LIM‐homeobox family among species. Lhx8 transcripts were detected in the neural crest‐derived mesenchyme of the first branchial arch at E9.5, and they are abundantly expressed in the dental mesenchyme at the bud stage (E12.5).^5^, ^6^, ^7^, ^8^ We previously found that Lhx8 regulates dental mesenchyme development as a negative gatekeeper of its differentiation and maturation.^9^ Further elucidating the detailed growth factors responsible for the dynamic change in Lhx8 is fundamental for clarifying tooth development.

On the other hand, the detailed target genes that Lhx8 regulates also remain evasive. As a transcription factor, Lhx8 could either activate or repress target gene expression via interacting with different partners. Recently, Lhx8 was found to interact with Suv39h1,^10^ which is a member of the suppressor of the variegation 3‐9 homolog family and encodes a protein with a chromodomain and a C‐terminal SET domain. Suv39h1 is a histone methyltransferase, methylating Lys‐9 of histone H3.^11^ It has been found to play a vital role in heterochromatin organization, chromosome segregation and mitotic progression.^12^ Recent results indicate that the histone H3 methylated on K9 is a binding site for HP1 family members, which in turn results in transcriptional repression.^13^ It is thus reasonable to deduce that the interaction between Lhx8 and Suv39h1 might promote cell growth and inhibit genes associated with differentiation simultaneously.

In this study, we employed a precise in situ hybridization technique to detect the expression pattern of Lhx8 and Suv39h1 throughout early murine tooth development. We then confirmed the dental epithelium‐mesenchymal signals’ possible regulation of Lhx8, and we explored the interaction between Lhx8 and Suv39h1, as well as the downstream effects ex vivo.

## 2 MATERIALS AND METHODS

### 2.1 Animal husbandry

Animal experiments were approved by the Animal Ethical and Welfare Committee of Sun Yat‐Sen University (Permit Number: 2018000056). All of the mice were housed under specific pathogen‐free conditions (22°C, 12‐hour light/12‐hour dark cycles, and 50%‐55% humidity) with free access to food and water. Three‐month‐old female C57Bl6 mice were mated with male mice, with the date of the vaginal plug appearance being Embryonic day 0.5 (E0.5).

### 2.2 Cell Culture

Proietics™ human Dental Pulp Stem Cells (DPSCs) were harvested from an adult third molar and cryopreserved in the primary passage (PT‐5025; Lonza, Alpharetta). DPSCs were maintained and expanded in Dulbecco Modified Eagle Medium (DMEM) at 37°C and 5% CO_2_. Cells were passaged at 80% confluence, with a medium change taking place every 2‐3 days.

### 2.3 Co‐Immunoprecipitation analysis

Co‐immunoprecipitation (co‐IP) analysis was performed with nuclear extracts. Briefly, nuclear lysates of DPSCs were supplemented with Protease Inhibitor Cocktail (Roche). The lysates were then centrifuged for 15 minutes at 12,000 g*,* and supernatant was collected. About 1 mg protein extract was incubated with 10 μg ChIP‐grade anti‐Suv39h1 monoclonal antibody (ab12405; Abcam) or 10 μg corresponding IgG control (ab172730; Abcam) for 12 hours at 4°C at a vertical shaking table. After that, 30 μL protein A Sepharose (ab193256; Abcam) was added for another 2 hours, followed by three washes with ice‐cold lysis buffer. The co‐immunoprecipitated complex was then subjected to Western blot analysis.

### 2.4 RNAscope in situ hybridization (ISH)

Tissue samples of mouse embryo or post‐natal mouse jaw were fixed in 10% neutral buffered formalin and were paraffin embedded according to standard protocols. ISH was performed on 5‐μm thick sections on a HybEZ Hybridization System using an RNAscope 2.5 HD Reagent Kit‐BROWN Kit (322300; Advanced Cell Diagnostics) or RNAscope 2.5 HD Duplex Reagent Kit (322430; Advanced Cell Diagnostics) according to the manufacturer's instructions. Sections were hybridized with mouse Lhx8 (300031; Advanced Cell Diagnostics), mouse Suv39h1 (489661; Advanced Cell Diagnostics), mouse Fgf8 (313411; Advanced Cell Diagnostics) and mouse Bmp2 (406661; Advanced Cell Diagnostics) probes. Briefly, the slides were dehydrated by using turpentine and 100% EtOH. Thereafter, the sections were treated with hydrogen peroxide solution for 10 minutes and then washed. Target retrieval was achieved at 100°C for 30 minutes. The slides were then treated with protease pre‐treatment solution for 30 minutes at 40°C in a HybEZ Oven. A hybridization probe was applied, and the slides were incubated for 2 hours at 40°C. After the wash and amplification steps, the signal was detected with DAB or Red/Green, counterstained and mounted. Images were acquired with Zeiss Image Z2 microscopy (Zeiss).

### 2.5 Lentivirus packaging and infection

For the overexpression of Lhx8 or Suv39h1, the open reading frames of target genes were amplified and the amplicon was inserted into the lentivirus expression vector of pWPI (Plasmid #12254; Addgene). A lentivirus was produced through transfecting HEK293T cells with the lentivirus expression vector along with pMD2.G (Plasmid #12259, Addgene) and psPAX2 (Plasmid #12260, Addgene) (pWPI 6.25 μg, pMD2.G 0.625 μg and psPAX2 3.125 μg). Supernatants containing lentivirus particles were then collected after 48 hours and were stored at −80°C before use. DPSCs were infected with a lentivirus in 8 μg/mL polybrene (Santa Cruz Biotechnology). DPSCs were transfected with the lentivirus or the negative control according to Multiplicity of Infection (MOI) 50:1 for 2 days.

For shRNA knockdown of Lhx8 or Suv39h1 in DPSCs, lentiviral shRNAs were purchased from GenePharma. Five shRNAs were used for lentiviral treatment. DPSCs were transfected with the lentiviral‐mediated shRNA or the negative control according to Multiplicity of Infection (MOI) 50:1 for 2 days. Compared with the non‐silencing scramble virus, the shRNAs with the highest knockdown efficiencies were chosen for follow‐up experiments. The chosen sequences were listed in Supplemental Table S1.

### 2.6 Cell seeding and in vivo transplantation

Prior to seeding, the 3D β‐tricalcium phosphate (β‐TCP) discs were pre‐wetted and sterilized with absolute ethanol for 30 minutes. Then, two 20‐minute washes were performed using sterile PBS, and a DMEM/F12 wash was performed for another 20 minutes. About 40 μL of the DPSCs suspension (1 × 10^7^ cells/mL) was injected onto each disc. After 3 hours for cell attachment, 12‐well culture plates containing 1 disc/well were filled with 2.5 mL of a complete medium/well. For in vivo animal experiments, the β‐TCP discs with DPSCs were implanted subcutaneously into the nude mice for 8 weeks as described earlier.^9^

### 2.7 Real‐Time Polymerase Chain Reaction (RT‐PCR) assay

Total ribonucleic acid (RNA) was extracted using Trizol (Thermo Fisher Scientific) from the E14.5 and E17.5 dental mesenchyme dissected under stereomicroscopy. cDNA was synthesized using Roche RT‐PCR System (Roche). Specific primers used for detecting mRNA transcripts are shown in Table S1. Transcripts were normalized to β‐actin or GAPDH and were compared with the control using the 2^‐ddCt^. Primers were listed in Supplemental Table S1.

### 2.8 Immunohistochemistry assay

Immunohistochemical (IHC) staining was done using HRP‐DAB Cell & Tissue Staining Kit (R&D Systems) according to the manufacturer's protocol. Briefly, paraformaldehyde‐fixed and paraffin‐embedded tissue samples were cut into 5 μm‐thick sections. Sections were deparaffinized, heat retrieved, blocked and thereafter incubated with the primary antibodies (DSPP, sc‐73632, 1:200, Santa Cruz Biotechnology) overnight at 4°C, followed by washing and incubation with HRP‐conjugated secondary antibodies. Signals were developed with DAB finally. The experiments were repeated at least 3 times.

### 2.9 CCK‐8 assay

To evaluate DPSCs’ proliferation ability, cells were seeded in 96‐well plates at a density of 1 × 10^3^ cells per well. DPSCs or DPSCs transfected with lentivirus were cultured with 100 ng/mL rhBMP2 (R&D Systems) or 25 ng/mL rhFGF8b (R&D Systems) every day. From day 1 to day 7, a 10 μL Cell Counting Kit‐8 (Vazyme) was used for cell proliferation evaluation by measuring the absorbance value at 450 nm.

### 2.10 EdU assay

An EdU (5‐ethynyl‐20‐deoxyuridine) assay was conducted using a Click‐iT Alexa Fluor 594 according to the manufacturer's protocol (Thermo Fisher Scientific). DPSCs or DPSCs transfected with lentiviral‐mediated shRNA were seeded on glass coverslips and cultured with 100 ng/mL rhBMP2 (R&D Systems) or 25 ng/mL rhFGF8b (R&D Systems) every day. On day 7 of culture, DPSCs were incubated with 10 μM EdU for 1 hour. Then, the cells were fixed in PFA, permeabilized with 0.1% Triton X‐100 and stained with Click‐iT Alexa‐594 dye‐conjugate for 30 minutes as instructed. Samples were co‐stained with Hoechst 33342 to visualize nuclei. Images were acquired using a LSM780 confocal microscope (Zeiss).

### 2.11 Transwell assay

The Transwell^®^ cell culture insert of an 8‐mm pore size (Corning, Corning) for a 12‐well plate was used. DPSCs were starved for 6 hours before seeding onto the insert at a density of 2 × 10^5^ in a DMEM basal medium with 0.5% foetal bovine serum (FBS, Thermo Fisher Scientific). Cells were subjected to migration induction cues (25 ng/mL rhFGF8b, 423‐F8, or 100 ng/mL rhBMP2, 355‐BM; R&D System) in a DMEM basal medium with a 0.5% foetal bovine serum in the lower chamber for 12 hours. The migrated cells in the lower chamber side of the insert membrane were fixed and stained by 0.1% crystal violet. Migrated cells were counted under the 10× light microscope, and the average number of 5 views/well was used to represent the migrated cell number.

### 2.12 Cell differentiation assay

DPSCs or DPSCs transfected with lentivirus were seeded at 60% confluence and were cultured with DMEM for 12 hours before switched to the odontogenic differentiation medium consisting of 100 μM ascorbic acid, 2 mM β‐glycerophosphate and 10 nM dexamethasone (Sigma‐Aldrich). The medium was changed every 2 days. For the treatment of BMP2 and FGF8, 100 ng/mL rhBMP2 (R&D Systems) and 25 ng/mL rhFGF8b (R&D Systems) were added every day for 7 days or else the indicated duration. RNA samples and protein samples were collected at the end of the experiments for gene expression analysis using RT‐PCR and Western blotting. Alizarin red staining was also performed at day 28 or otherwise indicated.

### 2.13 Western blot

Total protein was collected in RIPA Lysis Buffer (Cell Signaling Technology) with Protease/Phosphatase Inhibitor Cocktail (Sigma‐Aldrich). Proteins were separated on a NuPAGE^®^ Novex^®^ 4%‐12% Bis‐Tris Protein Gel (1.5 mm, 10 wells, Thermo Fisher Scientific) and transferred to the nitrocellulose membrane (MilliporeSigma). After blocking in 5% milk or bovine serum albumin (BSA), the following primary antibodies were applied overnight at 4°C: anti‐LHX‐8 (ab137036, Abcam), anti‐SUV39H1 (ab12405, Abcam), anti‐RUNX2 (12556, Cell Signaling Technology), anti‐DSPP (sc‐73632, Santa Cruz Biotechnology) and anti‐GAPDH (5174, Cell Signaling Technology) antibodies. The images were then developed, and the relative expression among different treatments was calculated.

### 2.14 Ex vivo organ culture

Mouse tooth germs at E12.5 were harvested and cultured ex vivo per conventional procedures.^14^, ^15^, ^16^ Briefly, the first mandibular molar tooth germs were dissected from E12.5 mouse embryo with fine forceps under a dissection microscope. The isolated tooth germs were cultured for up to 5 days with or without 100 ng/mL rhBMP2 (R&D Systems) and 25 ng/mL rhFGF8b (R&D Systems) ex vivo. The cultured tooth germs at day 5 were washed with cold PBS prior to gene expression analysis as described above.

### 2.15 Statistical analysis

Data were analysed with GraphPad Prism 7.0 or SPSS 22.0. Unpaired two‐tailed t test was used to compare the data of two groups. One‐way analysis of variance was used to compare the data of more than two groups. Data are presented as the mean ± SEM. Differences were considered to be significant when the *P* value was <0.05.

## 3 RESULTS

### 3.1 Lhx8 /Suv39h1 interaction inhibits odontogenesis

**FIGURE 1:**
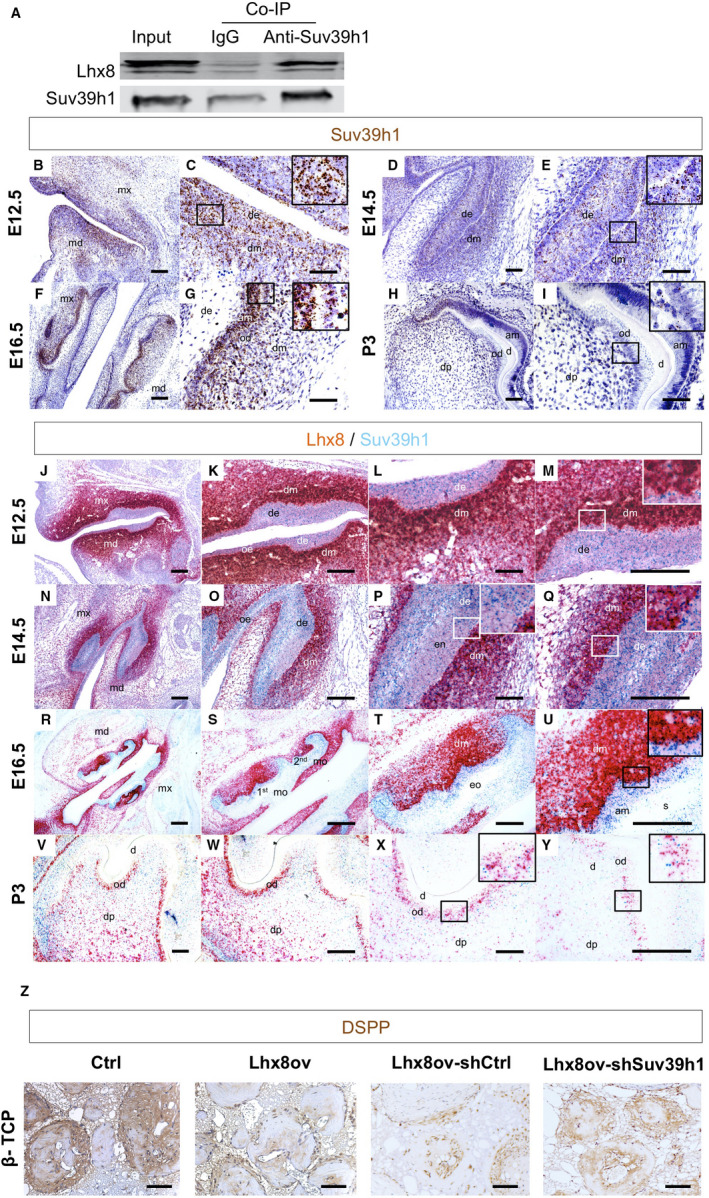
Co‐expression of Lhx8 and Suv39h1 regulating odontogenesis. (A) Co‐IP analysis of the interaction between Lhx8 and Suv39h1. (B‐I) Immunohistochemistry analysis of the expression of Suv39h1 in the tooth germ in E12.5 (B‐C), E14.5 (D‐E), E16.5 (F‐G) and P3 (H‐I). Representative data of five embryos/mice at each time point were shown. Suv39h1 was found to be ubiquitously expressed in both the dental epithelium and mesenchyme since E12.5, and it was significantly decreased in P3. (J‐Y) The in situ hybridization analysis of Lhx8 (red) and Suv39h1 expression (blue) in the tooth germ at E12.5 (J‐M), E14.5 (N‐Q), E16.5 (R‐U) and P3 (V‐Y). Representative images of 5 embryos/mice were shown. Suv39h1 and Lhx8 have different expression profiles, whereas both Lhx8 and Suv39h1 are abundantly expressed in the dental mesenchyme from E12.5 to E16.4. The DPSCs infected by an indicated lentivirus were seeded on the β‐TCP scaffold then subcutaneously implanted into nude mice to induce odontogenesis for 8 weeks. (Z) IHC staining of DSPP was shown. Representative images of 5 tests are shown. mx, maxillary process; md, mandibular process; de, dental epithelium; dm, dental mesenchyme; am, ameloblast; od, odontoblast; dp, dental papilla; d, dentin; oe, oral epithelium; en, enamel; mo, molar; eo, enamel organ; s, stellate reticulum. Scale bar: 100 µm

Consistent with previous findings,^10^ we here confirmed the interaction between Lhx8 and Suv39h1 in the DPSCs as revealed by a co‐IP assay (Figure 1A). For the purpose of further confirming the interaction in dental development, the expression of Suv39h1 and Lhx8 in a developing tooth was examined by using an RNAscope assay. Suv39h1 was found to be ubiquitously expressed in both dental epithelium and dental mesenchyme from E12.5 to E16.5, whereas it was significantly decreased in P3 (Figure 1B‐I). The co‐expression of Lhx8 and Suv39h1 in the dental mesenchyme from E12.5 to E16.5 (Figure 1J‐Y) was confirmed, suggesting that the interaction between Lhx8 and Suv39h1 occurs in vivo and thus should be functional during dental mesenchyme development. Further in vitro functional testing found that, when seeded in the β‐tricalcium phosphate discs, DPSCs overexpressing Lhx8 significantly down‐regulated the DSPP expression, whereas the additional knockdown of Suv39h1 significantly restored the DSPP expression (Figure 1Z). Together, the idea that Lhx8/Suv39h1 interaction during tooth development inhibits odontogenesis could be speculated.

### 3.2 Lhx8/Suv39h1 interaction inhibits odontogenesis via H3K9 methylation of target genes

We preliminary analysed the gene profile changes when Suv39h1 was knockdown in the DPSCs. Among the up‐regulated genes, 67 genes harboured the Lhx8 binding element (LBE) in their promoter region. Moreover, many of them were well‐known extracellular genes or transcription factors during odontoblast differentiation (data not shown). Furthermore, the ChIP assay that we conducted using the H3K9Me3 antibody showed that H3K9Me3 was enriched in the promoter regions of the selected genes in control DPSCs, whereas the enrichment was significantly decreased when either Lhx8 or Suv39h1 was knocked down (Figure S1).

### 3.3 Expression dynamic of the dental epithelium‐mesenchymal signals, Lhx8 and Suv39h1 in tooth development

**FIGURE 2:**
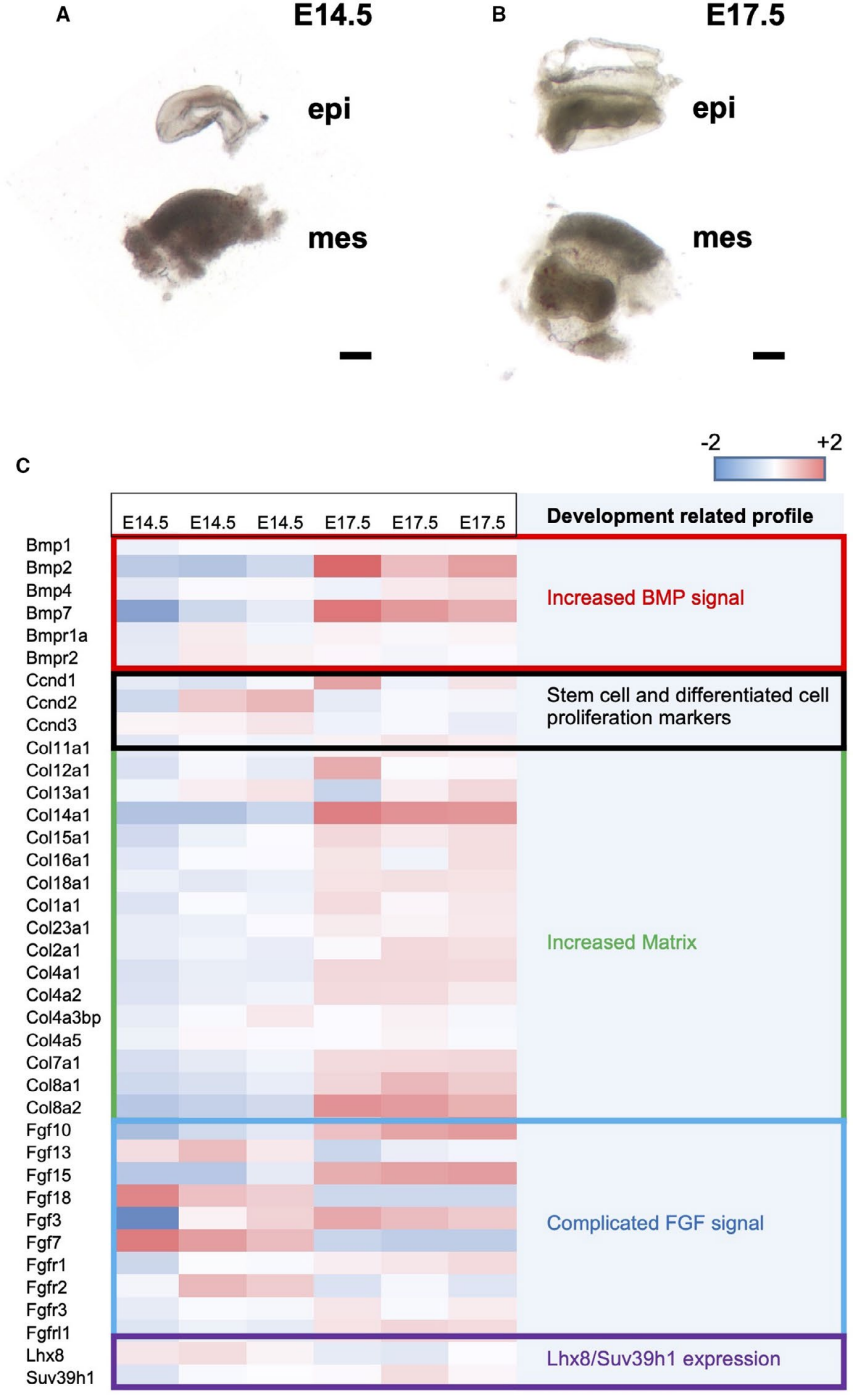
Profiling the expression of candidate genes in the developing dental mesenchyme. The representative samples of the dental mesenchyme at E14.5 (A) and E17.5 (B) dissected were shown. (C) Heatmap view of the expression of tested genes involved in cell differentiation and proliferation. epi, epithelium; mes, mesenchyme. Scale bar: 100 µm

The expression profile of the gene of interest gene in the dental mesenchyme at E14.5 and E17.5 was analysed by using RT‐PCR (Figure 2). Consistent with our previous findings, Lhx8 expression began to decrease at E17.5 with increased extracellular matrix genes and decreased proliferating markers, whereas Suv39h1 expression was stable from E14.5 to E17.5 (Figure 2C). Presumably, Lhx8, rather than Suv39h1, was subjected to the developmental regulation.

Bone morphogenetic protein 2 (BMP2) as a robust differentiation signal was highly expressed in the dental mesenchyme since E16.5 and peaks at P3 (Figure S2). In situ hybridization confirmed that Lhx8 was rarely expressed in P3 dental mesenchyme when Bmp2 was abundantly expressed (Figure S3). On the other hand, fibroblast growth factor 8 (FGF8) as a proliferation signal from the dental epithelium was highly and restrictedly expressed in the dental epithelium during early tooth development (Figure S4A,B). After E12.5, FGF8 gradually decreased and was rarely detected since afterwards (Figure S4C‐L). In situ hybridization analysis further confirmed that both Fgf8 and Lhx8 were expressed in the early tooth development, although in different regions, and they decreased with further tooth development (Figure S5). Together, these data suggested that the developmental dynamic of epithelium‐mesenchymal signals might be involved in the Lhx8 regulation and thus tooth development.

### 3.4 FGF8 promotes dental mesenchyme proliferation, whereas BMP2 promotes odontogenesis

**FIGURE 3:**
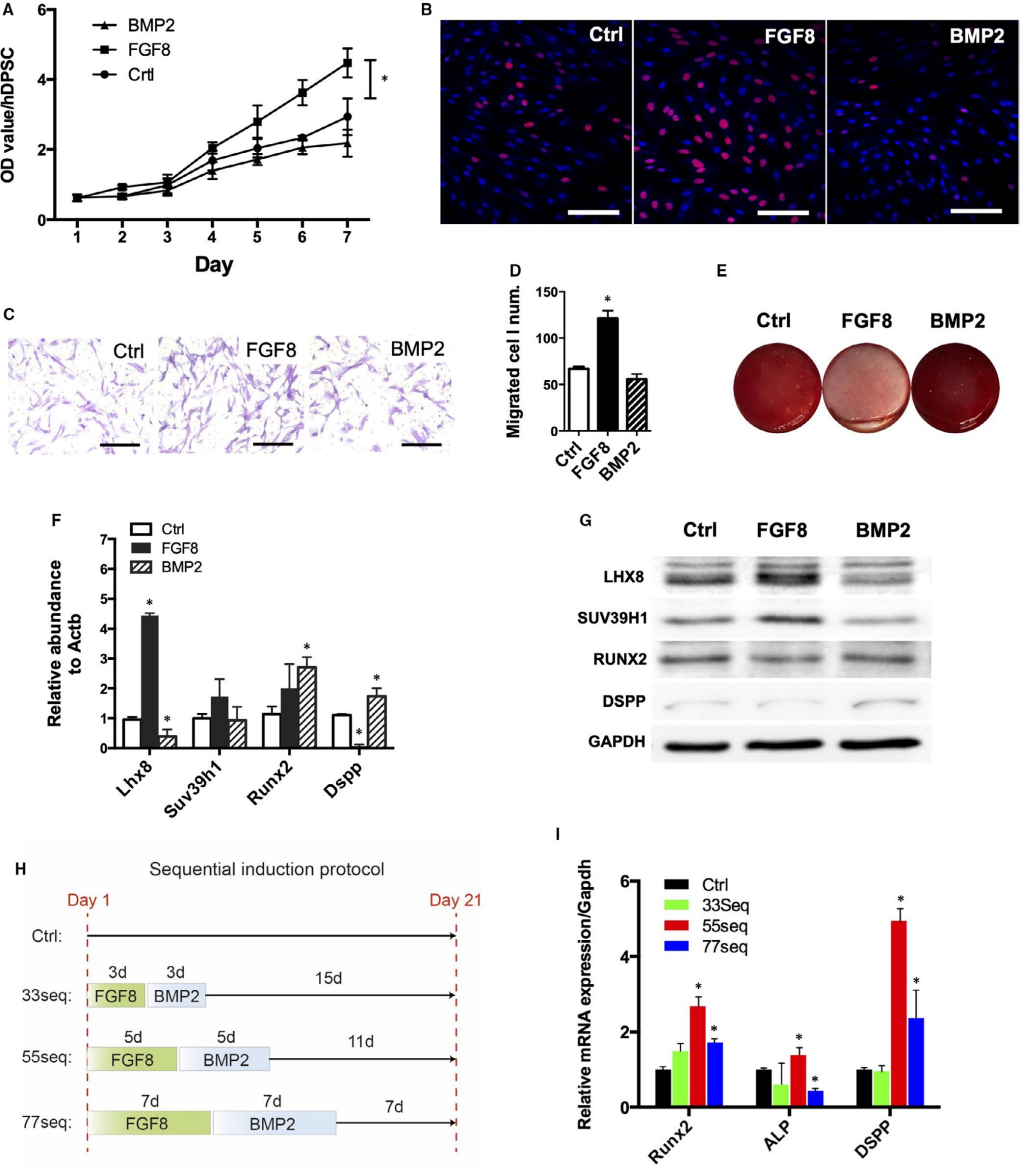
FGF8 and BMP2 regulate cell proliferation and differentiation in DPSCs. (A) CCK‐8 assay of DPSCs in the indicated treatment groups. Data were presented as mean ± SEM. *P < 0.05. (B) EdU staining of the proliferating DPSCs. Representative data of 3 independent experiments are shown. (C) Transwell assay of treated DPSCs (C), as quantified in (D). (E) Alizarin Red staining of the DPSCs of the indicated treatment groups. Representative data of 5 replicates were shown. (F) qPCR analysis of the expression of Lhx8 and selected genes upon FGF8 or BMP2 treatment. Data were presented as mean ± SEM. *P < 0.05. (G) Western blot assay of the protein expression of indicated genes in DPSCs treated with either FGF8 or BMP2. GAPDH served as the internal control. (H) Scheme of the sequential treatment procedures. (I) qPCR analysis of the odontogenesis‐associated genes of the sequential treatment groups. Data were presented as mean ± SEM. *P < 0.05. Scale bar: 100 µm

Consistent with the high expression of FGF8 in early tooth development (proliferating stages) and the high expression of BMP2 in the later tooth development (differentiating stages), FGF8 promoted DPSCs proliferation and migration, inhibited odontogenesis and activated Lhx8 expression (Figure 3A‐G). On the contrary, BMP2 promoted odontogenesis and inhibited Lhx8 expression (Figure 3A‐G). The sequential treatment protocols of FGF8 and BMP2 showed that the treatment of FGF8 for 5 days followed by another 5‐day treatment of BMP2 promoted odontogenesis most strikingly, as verified by the significantly increased gene expression of Runx2, Alp and Dspp (Figure 3H‐I).

### 3.5 FGF8/BMP2 regulates dental mesenchyme development via Lhx8/Suv39h1 complex

**FIGURE 4:**
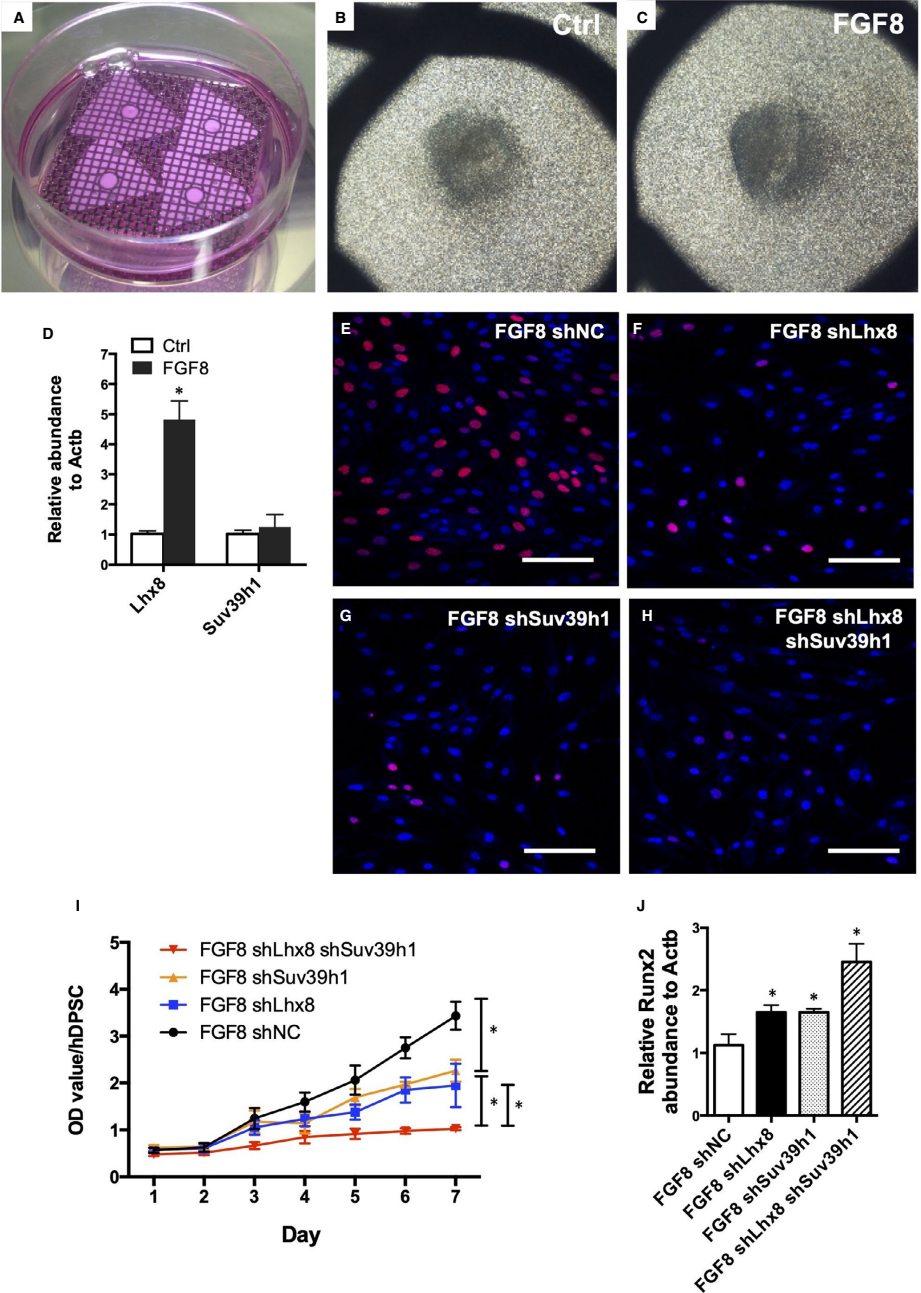
FGF8 regulates dental mesenchymal development via Lhx8/Suv39h1 complex. (A‐C) Organ culture of the dental mesenchyme treated with FGF8 for 5 days. (D) qPCR analysis of the expression change of Lhx8 upon FGF8 treatment. Data were presented as mean ± SEM. *P < 0.05. (E‐H) EdU staining of the DPSCs. DPSCs were infected by shLhx8 or shSuv39h1 or both lentiviruses, followed by FGF8 treatment and EdU incorporation. Representative data of 3 independent experiments are shown. (I) CCK‐8 analysis. Data were presented as mean ± SEM of three independent experiments. *P < 0.05. (J) qPCR analysis of Runx2 expression. Data were presented as mean ± SEM. * *P* < 0.05. Scale bar: 100 µm

Consistent with the cell culture experiments, the ex vivo organ culture model revealed that FGF8 significantly increased the expression of Lhx8 in the dental mesenchyme (Figure 4A‐D). The knockdown of any one or both of Lhx8 and/or Suv39h1 significantly repressed the cell proliferation on FGF8 treatment (Figure 4E‐I), whereas the expression of differentiation marker gene Runx2 was significantly enhanced (Figure 4J).

**FIGURE 5:**
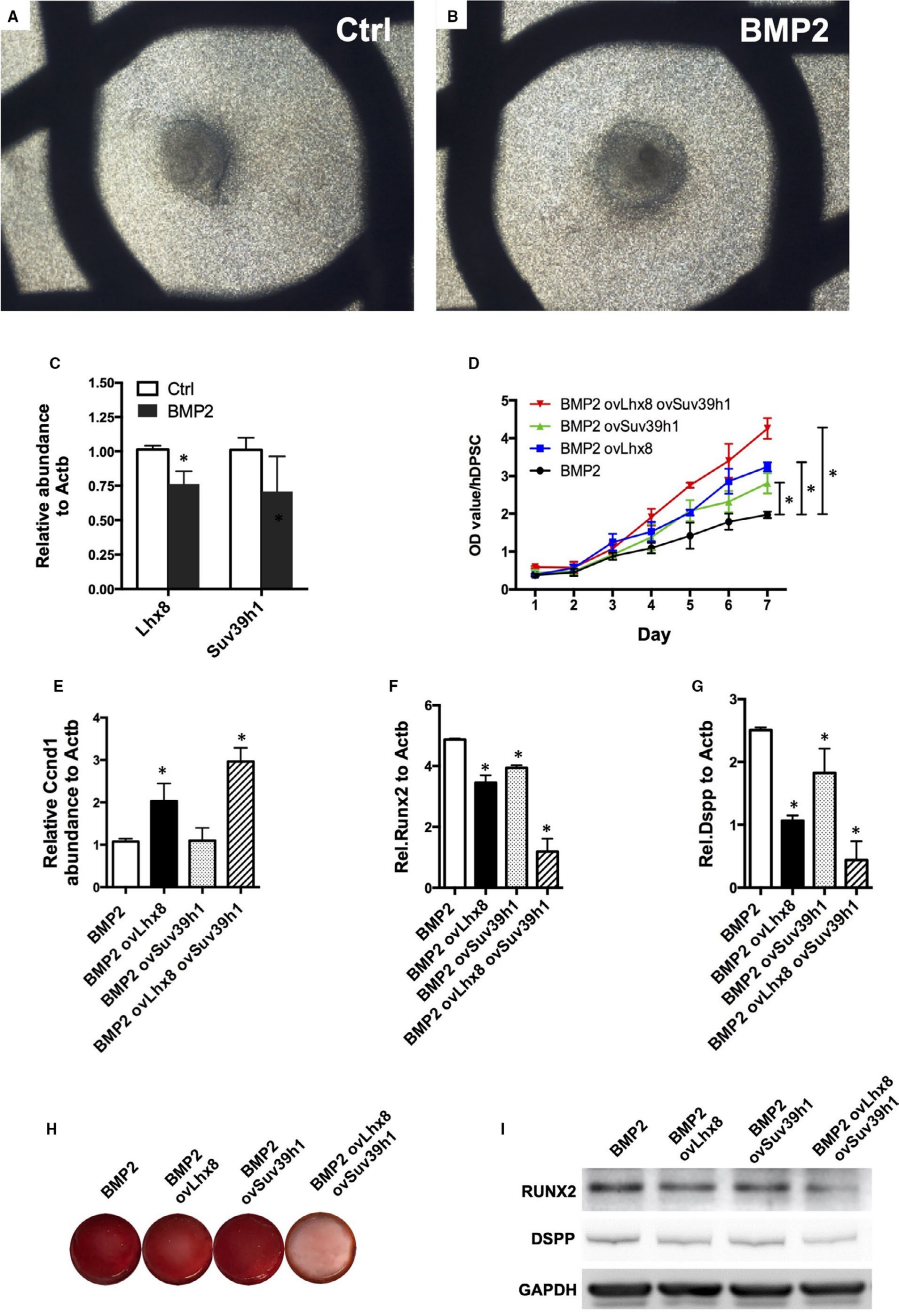
BMP2 regulates dental mesenchyme differentiation via Lhx8/Suv39h1 complex. (A‐B) Organ culture of the dental mesenchyme treated with BMP2 for 5 days. (C) qPCR analysis of Lhx8 and Suv39h1 upon BMP2 treatment. Data were presented as mean ± SEM. *P < 0.05. (D) CCK‐8 analysis. DPSCs were infected by ovLhx8 or ovSuv39h1 or both lentiviruses. (E‐G) qPCR analysis of Ccnd1, Runx2 and Dspp. Data were presented as mean ± SEM. *P < 0.05. (H) Alizarin Red staining. DPSCs were infected and treated with BMP2, followed by osteogenesis induction. Representative data of 3 independent experiments are shown. (I) Western blot assay of RUNX2 and DSPP

In contrast to FGF8, the expression of Lhx8 in the dental mesenchyme was found to be suppressed upon BMP2 treatment ex vivo (Figure 5A‐C). The overexpression of Lhx8 or Suv39h1 either alone or together significantly restored the repressed cell growth by BMP2 (Figure 5D,E). Moreover, overexpression of Suv39h1 or Lhx8 alone or together significantly reversed the effects of BMP2 on odontogenesis and proliferation (Figure 5F‐I).

## 4 DISCUSSION

Among the epithelium‐mesenchymal crosstalk signals, BMPs and FGFs are recognized as essential growth factors mediating inductive interactions throughout tooth development.^17^, ^18^, ^19^, ^20^ FGF and BMP signalling can regulate development antagonistically via targeting different transcription factors.^21^ Consist with these findings, the present study demonstrated that the dynamic change of FGF8 and BMP2 signals at least partially contributed to the expression profile of Lhx8. This, in turn, recognized the odontoblast differentiation‐related genes and repressed the gene expression via Lhx8/Suv39h1 complex‐mediated H3K9Me modification.

FGF8 was found to be highly expressed in the dental epithelium after E9.0^22^, ^23^ prior to the expression of Lhx8 in the dental mesenchyme. Before E14.5, no significant basement membrane exists between the epithelium and mesenchyme,^24^ making it possible for FGF8 in the dental epithelium to regulate the adjacent mesenchyme in a paracrine manner. Through the in vitro cellular model and organ culture, we confirmed the regulation of Lhx8 by FGF8. With tooth development, the dental epithelium‐mesenchyme crosstalk should be weaker as the basement membrane formed. To this end, a positive feedback loop might maintain the high expression of Lhx8.

BMP2/4/7 has shown differential spatial and temporal expression during the morphogenesis and odontogenesis.^25^ Previously, BMP4 was found to function as an inhibitor in the tooth site determination stage to restrict gene expression^26^ then as an activator to induce the expression of odontogenesis‐associated genes when tooth development occurs.^27^ Our study here further identified that BMP2 was rarely expressed in the early dental mesenchyme, whereas it was highly expressed in the later stages after E16.5. The gene expression of BMP2 at later stages of tooth development is consistent with the role of BMP in promoting odontogenesis.^28^, ^29^, ^30^ Besides the known targets of BMP2, we further identified that BMP2 repressed Lhx8, although the detailed mechanism for how Lhx8 was down‐regulated warrants further study. In any case, our work here further revealed that the dynamic expression of FGF8 and BMP2 fine‐tunes the dental mesenchyme development during embryonic and post‐natal tooth development, and the biological effects largely rely on the expression of Lhx8.

Together, in the early stages, FGF8 induces Lhx8 and appears to maintain dental mesenchymal cells in an undifferentiated stage via H3K9 methylation in the promoter regions of multiple differentiation‐associated genes and subsequent gene expression. There are putative Lhx8 binding sites in the promoters of all the genes of interest. The histone modification usually spreads in the promoter region after initiation.^31^ In this study, we just explored the binding of H3K9Me3 in the core promoter region, which is considered as essential for the transcriptional repression. The transcriptional repression of these differentiation genes allows for proliferation. With development, accumulating BMP2 decreases Lhx8 expression gradually. Vanishing Lhx8 in the later stage releases Suv39h1 from the promoters, which then promote odontoblast differentiation and dentin formation (Figure S6). The present study has set a good example for how a growth‐promoting gene inhibits cell differentiation simultaneously. Ongoing studies using conditional Lhx8 and/or Suv39h1 knockout mice would further confirm the stage‐specific roles of Lhx8 in tooth development.

## CONFLICT OF INTEREST

The authors declare that they have no conflict of interest.

## AUTHOR CONTRIBUTIONS

**Chen Zhou:** Conceptualization (equal); Formal analysis (equal); Funding acquisition (lead); Investigation (equal); Methodology (equal); Writing‐original draft (equal). **Danying Chen:** Formal analysis (equal); Investigation (equal); Methodology (equal); Writing‐original draft (equal). **Jianhan Ren:** Formal analysis (supporting); Investigation (supporting). **Delan Huang:** Formal analysis (supporting); Investigation (supporting). **Runze Li:** Formal analysis (supporting); Investigation (supporting). **Haotian Luo:** Formal analysis (supporting); Investigation (supporting). **Chenyu Guan:** Formal analysis (equal); Validation (equal). **Yang Cao:** Resources (equal); Supervision (equal). **Weicai Wang:** Project administration (lead); Supervision (supporting); Writing‐review & editing (lead).

## ETHICAL APPROVAL

All procedures performed in the present study involving humans and animals were in accordance with the ethical standards of the Animal Ethical and Welfare Committee of Sun Yat‐Sen University (Permit Number: 2018000056).

## Supporting information

Figure S1

Figure S2

Figure S3

Figure S4

Figure S5

Figure S6

Table S1

## Data Availability

The data that support the findings of this study are available in the supplementary material of this article, and also available on request from the corresponding author.
