# Supplementary figures and images for: FGF8 and BMP2 mediated dynamic regulation of dental mesenchyme proliferation and differentiation via Lhx8/Suv39h1 complex
Source: J Cell Mol Med. 2021 Feb 13;25(6):3051–62. doi: 10.1111/jcmm.16351 (PMC7957265; doi:10.1111/jcmm.16351)

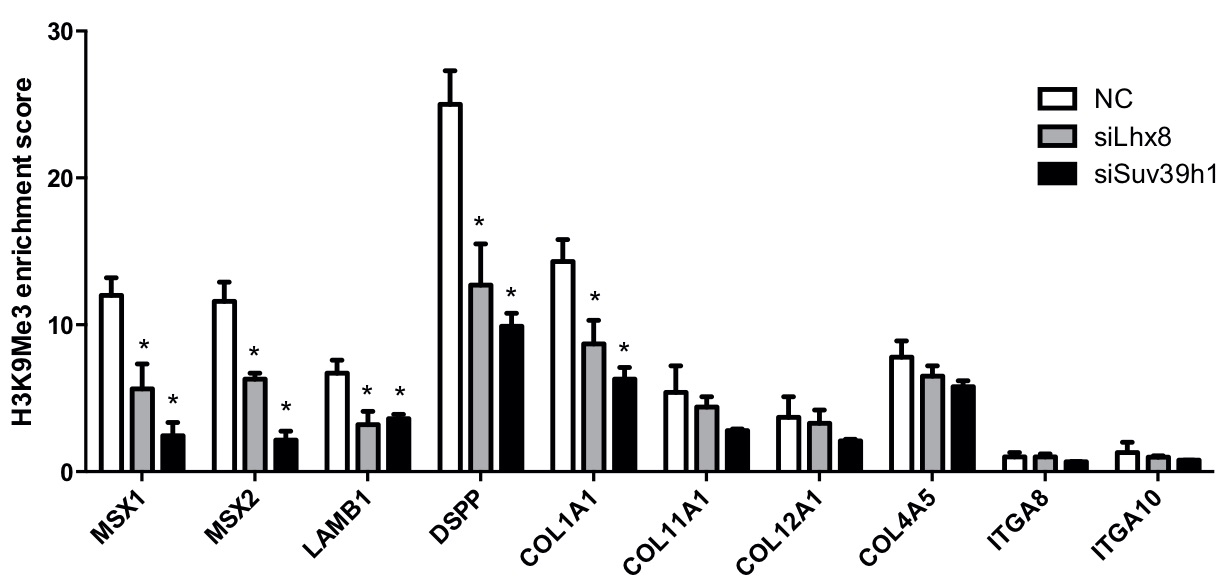

Supplement: Supplementary file 1 — Figure S1 [file JCMM-25-3051-s002.jpg]

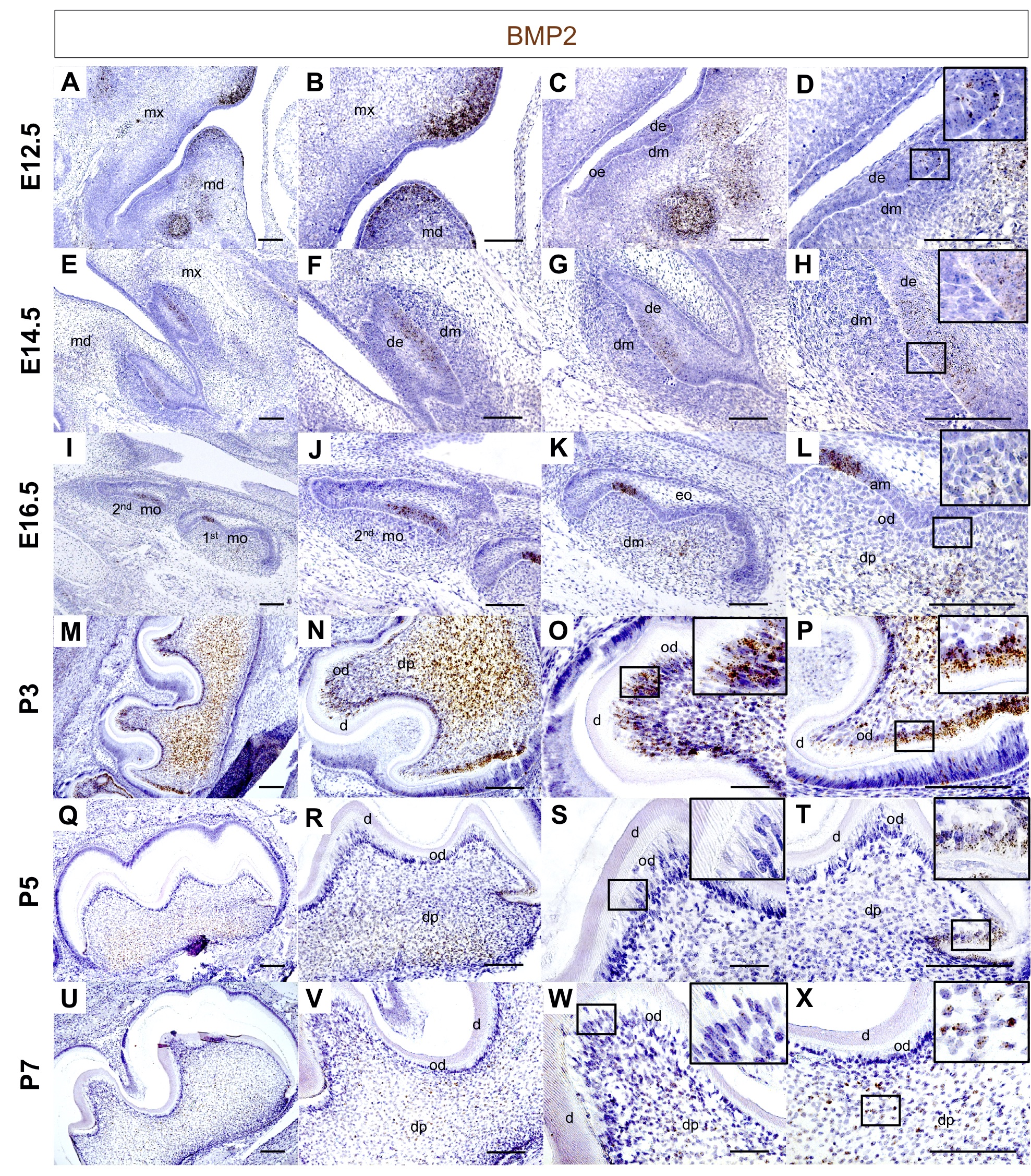

Supplement: Supplementary file 2 — Figure S2 [file JCMM-25-3051-s004.jpg]

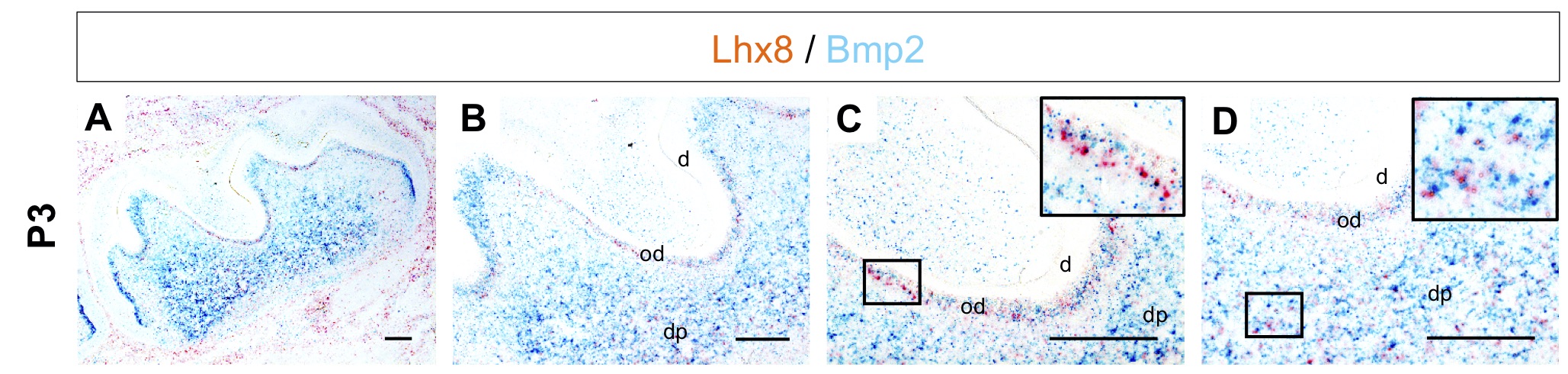

Supplement: Supplementary file 3 — Figure S3 [file JCMM-25-3051-s003.jpg]

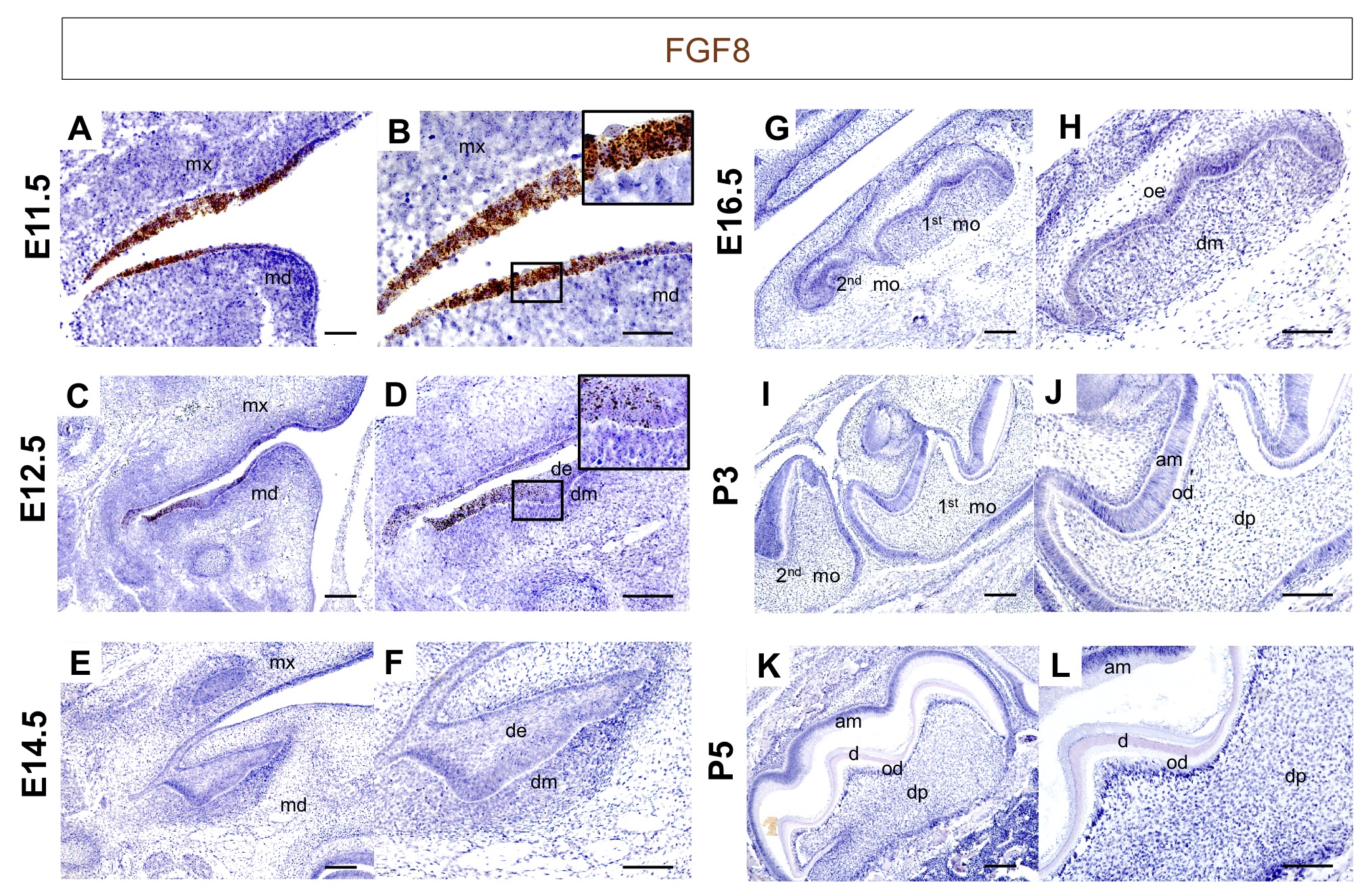

Supplement: Supplementary file 4 — Figure S4 [file JCMM-25-3051-s005.jpg]

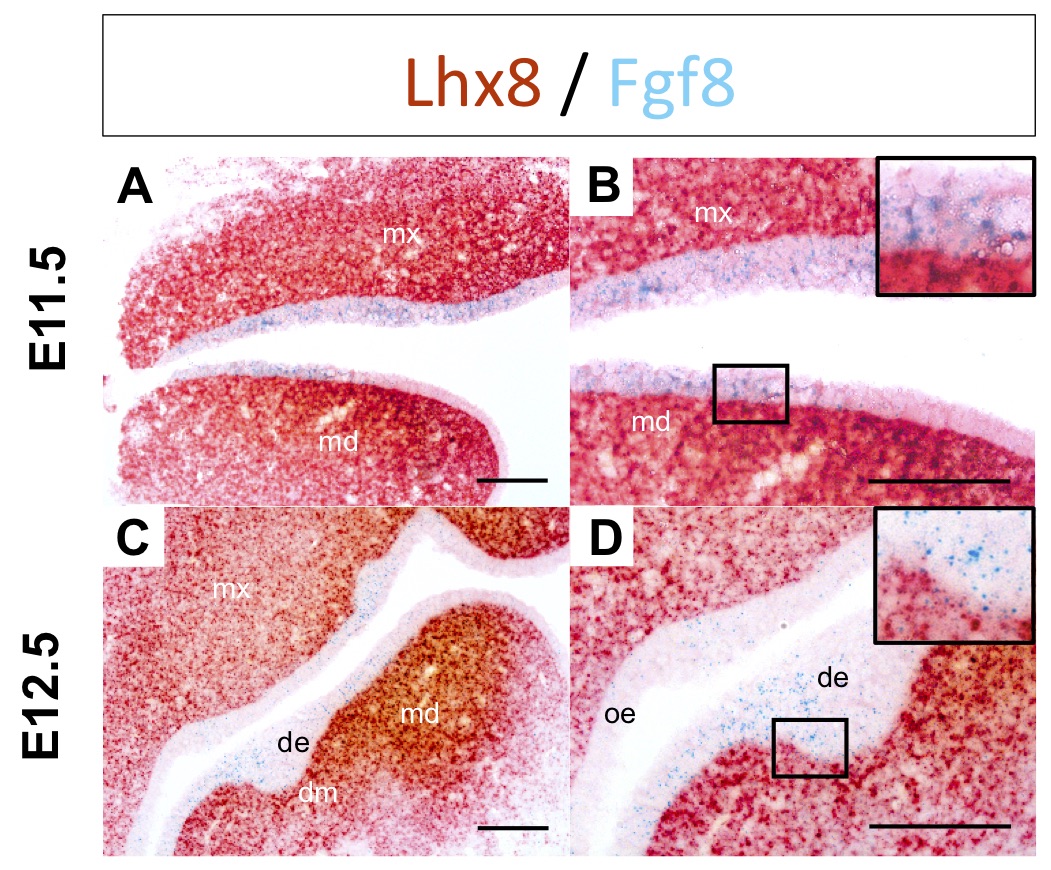

Supplement: Supplementary file 5 — Figure S5 [file JCMM-25-3051-s001.jpg]

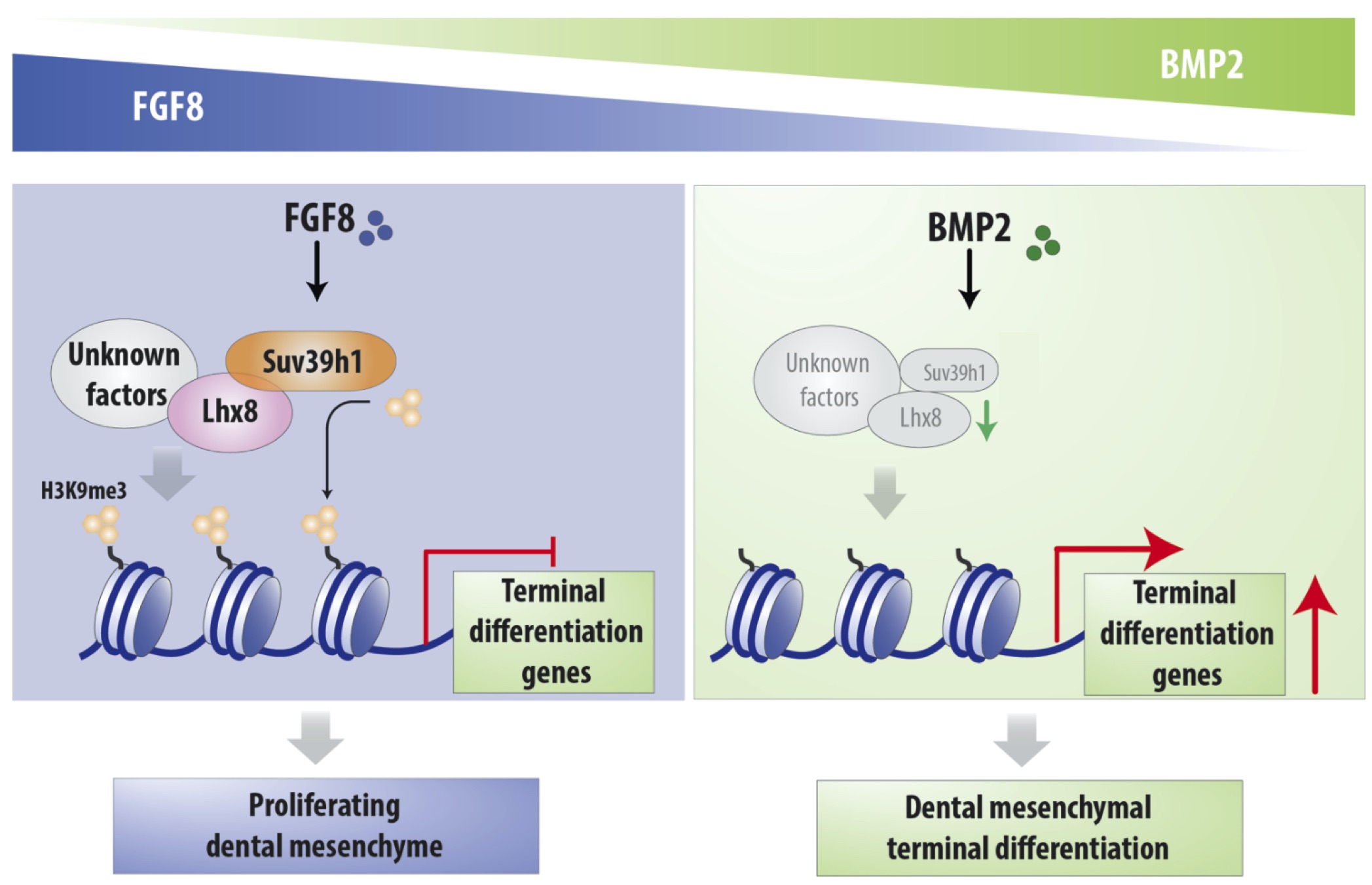

Supplement: Supplementary file 6 — Figure S6 [file JCMM-25-3051-s007.jpg]
